# A Microfluidic Experimental Method for Studying Cell-to-Cell Exosome Delivery–Taking Stem Cell–Tumor Cell Interaction as a Case

**DOI:** 10.3390/ijms241713419

**Published:** 2023-08-30

**Authors:** Xing Yue (Larry) Peng, Pengxiang Su, Yaxin Guo, Jing Zhang, Linghan Peng, Rongrong Zhang

**Affiliations:** Biology Department, Xiamen University, Xiamen 361102, China

**Keywords:** cell–cell communication, exosome, microfluidic culture dish, stem cell, metastasis, niche, microcirculation

## Abstract

Cell-to-cell communication must occur through molecular transport in the intercellular fluid space. Nanoparticles, such as exosomes, diffuse or move more slowly in fluids than small molecules. To find a microfluidic technology for real-time exosome experiments on intercellular communication between living cells, we use the microfluidic culture dish’s quaternary ultra-slow microcirculation flow field to accumulate nanoparticles in a specific area. Taking stem cell–tumor cell interaction as an example, the ultra-slow microcirculatory flow field controls stem cell exosomes to interfere with tumor cells remotely. Under static coculture conditions (without microfluidics), the tumor cells near stem cells (<200 µm) show quick breaking through from its Matrigel drop to meet stem cells, but this ‘breaking through’ quickly disappears with increasing distance. In programmed ultra-slow microcirculation, stem cells induce tumor cells 5000 μm far at the site of exosome deposition (according to nanoparticle simulations). After 14 days of programmed coculture, the glomeration and migration of tumor cells were observed in the exosome deposition area. This example shows that the ultra-slow microcirculation of the microfluidic culture dish has good prospects in quantitative experiments to study exosome communication between living cells and drug development of cancer metastasis.

## 1. Introduction

All cells within the body begin as stem cells, and communication between cells plays a significant role in deciding their fate. Cell-to-cell communication [1] involves the transmission of a signal from a sending cell to a receiving cell, and this process may be reciprocal. Cell-to-cell communication involves life processes and can even induce cell death [2]. Exosomes are a subset of extracellular vesicles (EVs) of endosomal origin and in a size range of ~40 to 160 nm in diameter (~100 nm on average), containing nucleic acids, proteins, lipids, amino acids, and metabolites [3]. Exosomes are the primary way in cell-to-cell communication. Exosomes have shown therapeutic potential as a drug or vaccine delivery system [4] and serve as cell-to-cell communication modulators in healthy and diseased brains [4]. Regarding physical properties, exosomes as EVs can be divided into subclasses according to sedimentation characteristics or size [5], for example, >300 nm by centrifugation around 2000× *g*, and the smallest <150 nm EVs by around 20,000× *g*. But regardless of size, exosomes function through the molecules they transport (nucleic acids, proteins, lipids, amino acids, and metabolites). For example, nucleic acids can be used as disease markers and intercellular communication molecules [6].

The questions about exosomes focus on understanding their constituents’ fate and the phenotypic and molecular alterations that they induce in recipient cells in cell-culture systems [3]. In fact, as EVs, exosomes range in diameter from tens of nanometers up to several micrometers and can transfer biologically active cargoes from whole organelles, through macromolecules including nucleic acids and proteins, to metabolites and small molecules, from their cells of origin to recipient cells [7]. This packaging and transport of exosomes ensures that their contents are not easy to diffuse during transportation so that the molecules can act at longer distances. Therefore, studying the dynamics of exosome packaging and transport and accurately quantifying packaged molecules’ long-range action mechanisms is essential. At the same time, those soluble molecules that are not packaged, such as soluble intercellular adhesion molecules [8], also participate in cell-to-cell communication. Studying the detailed process of exosomes in cell–cell communication faces significant technical challenges because the transfer process of exosomes between living cells cannot be directly controlled, and only exosomes can be extracted for experiments. However, extracting exosomes will bring great uncertainty to the investigation. The potential inconsistencies in identifying regulatory elements associated with exosome biogenesis could also result from different methods for exosome production, enrichment, and concentration [9]. In addition to separation from EVs by centrifugal precipitation [5], exosomes can be subdivided according to size, content, function, and source heterogeneity [3]. Exosome secretion kinetics [8] have also been studied to a certain extent. The velocity distribution during exosomes’ motion on the cell surface is also measured more accurately (about 100 nm s^−1^).

The following questions capture the difficulty in studying cell-to-cell communication: (A) How do we properly separate cells and control signal molecules’ transmission direction and time? (B) How do we prevent a small number of signal molecules produced by cells from being diffused and lost during long-distance transmission? (C) How do we distinguish the role of EVs from soluble molecules? (D) How do we simulate the cellular microenvironment to obtain artificial microcirculation without losing information molecules? Simultaneously achieving the goals of A–D above will require new technologies. Based on the unique magnetic micropump technology [10] and the microfluidic culture dish technology [11], we will find a way to solve the above A–E difficulties at the same time. We will take the stem cell–tumor cell interaction as a case [12] to investigate the characteristics of the new technology and the detailed process in cell-to-cell communication research.

For the above difficulties A–E, our thinking is as follows: (A) use microfluidic technology in a culture dish [10] to control the transmission direction and time of signal molecules (EVs); (B) precisely control the pattern and speed of the circulating flow, which allow EVs to be deposited in a fixed area; (C) distinguish the difference between the diffusion of soluble molecules and the distribution field of EVs; (D) use the technology of precise ultra-slow microcirculation cell nests in microfluidic culture dishes [11] to solve the problem of nutrient supply and maintenance of the cell microenvironment; and (E) place cells in Matrigel in the upstream and downstream of the ultra-slow microcirculation (that is, the starting point and the endpoint area of EVs). Exogenous bolus doses of supraphysiological levels of cancer exosomes in mice were associated with the induction of neoplasia [13]. Stem cells, including embryonic and adult stem cells, are special human cells that can develop into many different cell types. Human induced pluripotent stem (hiPS) cells are a type of pluripotent stem cell derived from human adult somatic cells, similar to embryonic stem (ES) cells in many aspects. The stem cell–tumor cell interaction model was chosen because tumor metastasis and the activity of stem cells are positively correlated [12]. The literature and our experimental data also indicate that stem cells may trigger the progression and metastasis of tumor cells through EVs. For example, the stem cell niche may promote metastasis [14] or accelerate tumor progression [15] because the bone marrow, where mesenchymal stem cells are abundant, is the most common place of fatal metastasis because of stem cell induction [15]. Stem cell exosomes in patients with multiple myeloma promote cancer cell growth [16]. Stem cell accumulation in the inflammation site [17] or the wound site [18] might explain the cancer metastasis there.

The stem cell–tumor cell coculture is carried out in a microfluidic culture dish. The purpose is to explore a general experimental technique to precisely control cell–exosome–cell communication and apply this experimental technique to stem cell–tumor cell interaction research to explore the use and advantages of this new technology. Exosomes with different immunophenotypes have biomarkers (CD9/CD63/CD81) [3]. This technology may allow cell–exosome–cell parts to be spatially separated for intercellular communication experiments. In the future, it can be expected that the transfer of exosomes between cells can be tracked in real-time with immunofluorescence biomarkers.

## 2. Results

### 2.1. The Artificial Cell Nest for Transport and Deposition Modes of Nanoparticles

**Figure 1:**
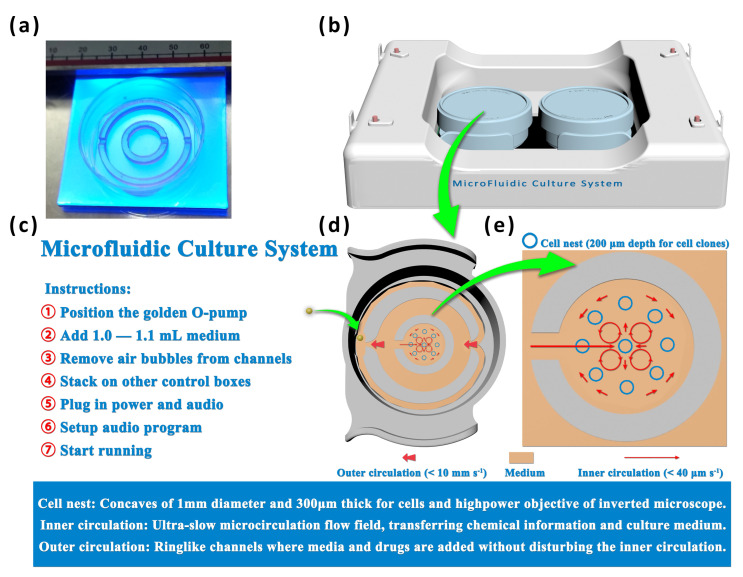
Microfluidic culture dish system. (**a**) Microfluidic culture dish made of polycarbonate (PC, sterilizable at 105 degrees) or polystyrene (PS). (**b**) Microfluidic culture made by injection molding process microfluidic culture dish system (can control two microfluidic culture dishes). (**c**) Operation steps of the microfluidic culture dish. (**d**) The internal structure of the microfluidic culture dish and the magnetic control micropump can be added or removed (small golden ball). (**e**) The central cell nest’s ultra-slow microcirculation quaternary flow field in the microfluidic culture dish (red arrow).

**Figure 2:**
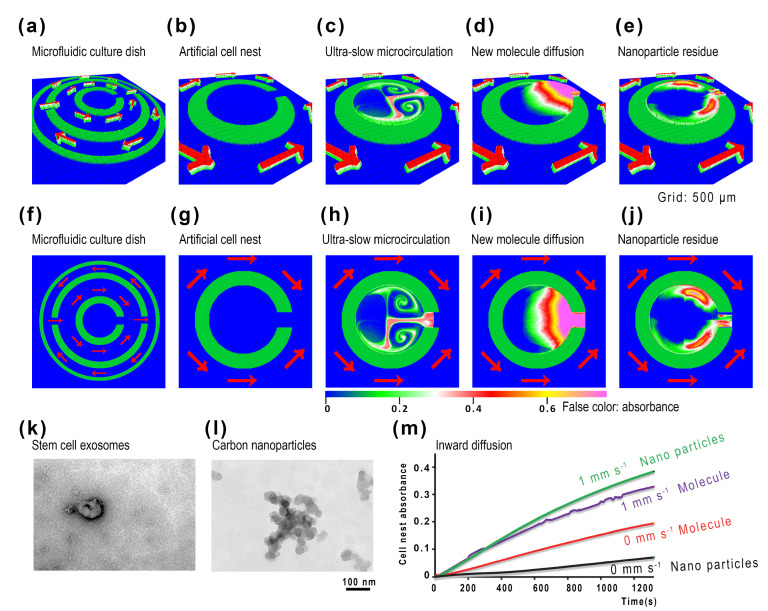
The ultra-slow microcirculation, nanoparticle residue, and molecule diffusion in the artificial cell nest. There is a controlled circulation in the microfluidic culture dish. (**a**) When the circulation flows through the central cell nest (**b**), an ultra-slow microcirculation (carbon nanoparticle indication) appears (**c**) in the central cell nest. The molecules diffuse into the nest like a fan (**d**). The nanoparticles in the nest have residual areas on the edges and both sides of the doorway (**e**). The 2D images of (**a**–**e**) are (**f**–**j**). The exosomes of stem cells (**k**) and carbon nanoparticles (**l**) have similar diameters. When the outer circulation stops and flows, the rate at which carbon nanoparticles and molecular ink (C_20_H_6_Br_4_Na_2_O_5_, MW 691.85) enter the nest becomes significantly different (**m**). The photo of a microfluidic culture dish is shown (Figure 1) (See Video S1).

The microchannels of the microfluidic culture dish [11] (Figure 1) control the flow direction of the microfluidics (Figure 1e). It drives fluid outside the central cell nest (Figure 2a,f) through a semiclosed cell nest (Figure 2b,g). The flow inside the cell nest has ultra-slow microcirculation (Figure 2c,h, <40 μm s^−1^). The high diffusion molecules at the gate show a fan-shaped gradient (Figure 2d,i). However, the low diffusion nanoparticles penetrate deep into the artificial cell nest along the circulation and eventually deposit on both sides of the gate (Figure 2e,j). The nanoparticles of similar size to exosomes (Figure 2k,l) represent exosomes’ distribution. Molecular diffusion also represents most molecules’ exchange rate. Figure 2m shows the fluxes of nanoparticles and molecules with or without microflow.

From Figure 2c,d or Figure 2h–j (see Videos S1 and S2), we can see that nanoparticles and molecular substances enter the C-type cell nest differently. Nanoparticles (represented by carbon nanoparticles as EVs) diffuse slowly and can form a 100–200 μm wide quaternary microflow in the cell nest along with the ultra-slow microcirculation (Figure 2c,h). These nanoparticles move with the flow and form a stable upstream–downstream relationship at different positions inside the entire cell nest. For example, the inner two vortices in the circulation flow near the inside are located upstream, while on both sides of the gate, the outer two vortices are located downstream. Exosomes secreted by cells deep inside will eventually reach both sides of the doorway (Figure 2e,j, see Videos S1 and S2). Molecules in the medium, however, diffuse much faster than nanoparticles. When these molecules enter the cell nest with the microflow (<40 μm s^−1^), they begin to diffuse to both sides (Figure 2d,i, see Video S1). Since the flow velocity of the liquid (medium) is slower than the diffusion velocity of the molecules, the actual diffusion pattern of the molecules is not affected by the shape of the flow field (Figure 2d,i). These molecules will form a concentration gradient at the gate, and the nanoparticles will be carried away by the circulation and deposited on both sides of the doorway (Figure 2e,j). The accumulation patterns on both sides of the doorway are entirely different (compare Figure 2i with Figure 2j, Videos S1 and S2).

Nanoparticles are similar in size to EVs (Figure 2k,l), so the EVs released by the stem cells inside the cell nest will show an equal distribution and deposition to nanoparticles due to the physical characteristics of EVs that are not easy to spread [8]. Exosomes have been observed to diffuse far from the site of secretion [19] or remain close to the cell surface [20,21]. Because of the ultra-slow and delicate microcirculation, this long-distance migration of EVs can reach a millimeter scale without much loss. This technology gives us many in vitro experimental choices for cutting off or programming control of EVs. At the same time, the determined internal and external exchange rate (molecular or nanoparticle delivery) is also conducive to our precise experimental design. The microfluidic coculture of living cells can also be used for drug testing or nutrient supply.

### 2.2. The Stem-Cell-to-Tumor-Cell Co-Culture without Microfluidics

Cell-to-cell communication is mainly carried out through EVs (exosomes). But exosomes diffuse very slowly, and the distance they can travel without flow is minimal. And because the diffusion is nondirectional, the concentration of exosomes decays rapidly with the increase of space. Although a coculture without flow is not convenient for an in depth study of cell-to-cell communication (because all chemical molecules need to be controlled by microflow), this experiment can at least help us understand the effective distance of cell-to-cell communication.

**Figure 3:**
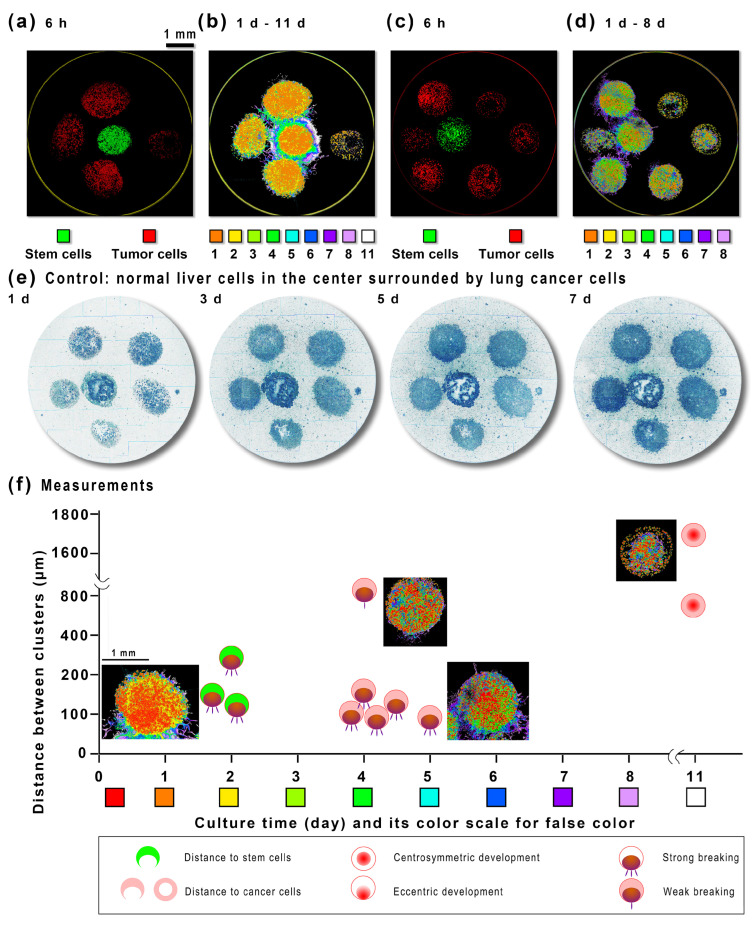
The stem cell–cancer cell close-range interaction in the simulated matrix. (**a**) A stem cell (iPSC) Matrigel drop (green) and four cancer cell Matrigel drops (red) covered by 20× Matrigel in a 6mm circular dish. (**b**) The color scale showed the developments and the cell invasions from the mimic tumors in 1–11 days. (**c**) Another stem cell Matrigel drop (green) and six cancer cell Matrigel drops (red) covered by 20× Matrigel in a 6mm circular dish. (**d**) The developments and the cell invasions from the mimic tumor in 1–8 days. (**e**) As a control, normal liver cells (L-02) in the center, surrounded by five cancer cell clusters in 7 days of co-cultivation. (**f**) The developments and the invasion types of the mimic tumors plotted on the distance to stem cells or other cancer cells. The distance between the cell clusters is between the nearest edges measured in the first 24 h. The typical small images next to the symbol are parts of the full images.

We mixed cancer cells or stem cells in 5× Matrigel drops to act as mimic tumors, planted them on the bottom of the 6 mm 96-well plate (Figure 3a), and covered them with 20× Matrigel. The Matrigel slowed the molecular diffusion to form an inducer gradient or a stem cell niche. Far from the stem cells (>500 μm), the development of mimic tumors was centrosymmetric, with no evident effect from the stem cells (Figure 3b). If mimic tumors are closer (200–600 μm) to the stem cells, the mimic tumor development is excentric in the adjacent stem cells’ direction. The cancer cell clones proliferate to over 50 μm in diameter in two days and break the mimic tumor’s border in four days. If the distance is shorter than 200 μm, the breaking of mimic tumors becomes energetic, and the cells go out of their matrix drop to meet the cancer cells. This distance-dependence invasion indicates that the stem cells have formed a circular induction force around them. By further adjusting the distance between each cell cluster (Figure 3c), this result was repeated in Figure 3d. As a control, we replaced the central cell cluster with a normal liver cell cluster (Figure 3e), surrounded by five A549 cell clusters (mimic tumors). In the 7-day coculture under the same conditions, the interaction between the central liver cell cluster and the surrounding A549 cell clusters and the interaction between the surrounding A549 cell clusters are not significant. No active cells are found in the area between the cell clusters. Although the cells in the matrix glue of each cell cluster are still growing and proliferating (Figure S2), the cell cluster junction area is far less intense than the stem cell–tumor cell junction area when there is a stem cell cluster in the middle (Figure 3d). For the relationship between stem cell–tumor cell distance and cell cluster development type (Figure 3f), we can see that within 100–200 µm, stem cells induce tumor cells within two days, while tumor cells need at least four days to do that. The induction effect is insignificant if the distance is too far or there are only normal stem cells at the center (see Video S3).

### 2.3. The Microfluidic Co-Cultivation of Stem Cells and Tumor Cells in Typical Culture Dishes

**Figure 4:**
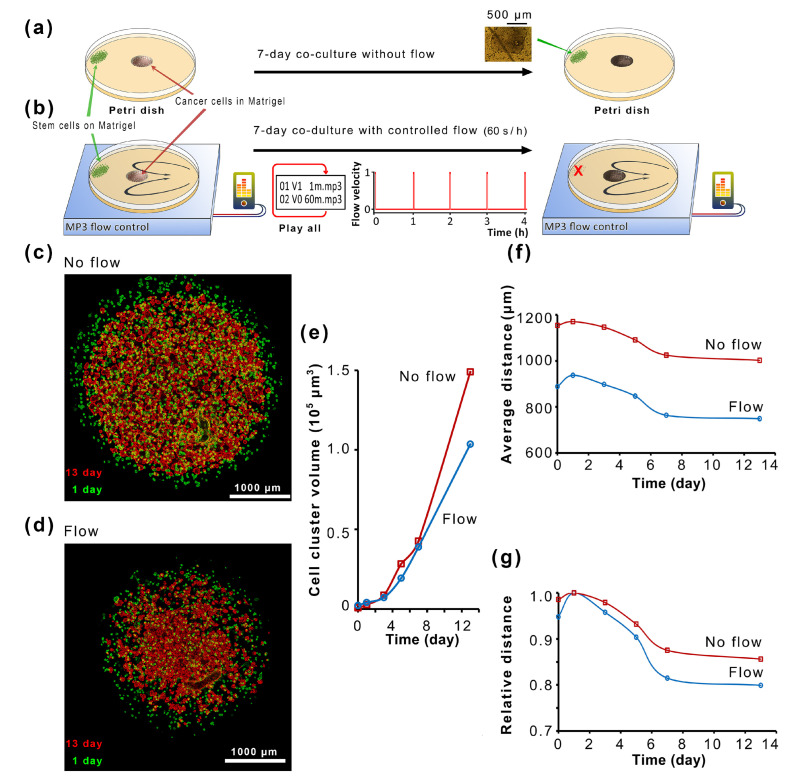
Microfluidic co-cultivation of stem cells and tumor cells in typical culture dishes. We plant stem cell clones (hiPSC) and a mimic tumor (A549) in regular culture dishes (**a**) and control their flow (black arrows) with an MP3 player (**b**). The flow program is set by editing the playlists and setting a loop of 60 s flow per hour. We record 13-day images and merge them to color-time scale false-color images (**c**,**d**). Each cancer cell clone is measured for diameters and positions. Then we calculate (N > 1000) the average cell clone volumes (**e**) and average radius (**f**) or relative average radius (**g**). The average radius formula is $\bar{R}=\frac{\sum V_{i}R_{i}}{\sum V_{i,}}$, where *R_i_* is the radius of an individual cell clone, and Vi is its volume.

By controlling the microflow, it is possible to control the path and direction of EVs so that more experiments can be performed. An easy way to perform this is to let the medium flow in a typical culture dish. The O-pump [10] is a micropump that can be dropped directly into a culture dish. As shown in Figure 3, stem cells can be co-cultured with tumor cells in Matrigel droplets. After confirming that both cells survived and started proliferating (Figure 4a,b), we start the flow program (Figure 4b).

In contrast, the other group added an O-pump and drove the flow program in the culture dish to run (1 min per hour). There is no microchannel design in typical culture dishes, and the O-pump produces a butterfly-shaped flow field (Figure 4a) [11], and the flow velocity attenuates (0–5 mm s^−1^) with the distance of the O-pump. After seven days, the stem cells grew as 0.5 mm colonies in culture dishes without flow (Figure 4a). In a culture dish with the flow, stem cell clones disintegrate. This is because the large-scale and too-strong flow will cause damage to the microenvironment of stem cells and apoptosis of stem cells. Therefore, while using microfluidics to provide nutrients for stem cells, it is necessary to consider how to update microfluidic technology to maintain the microenvironment of stem cells. For tumor cells, because they are protected by droplet-shaped Matrigel, their growth inside Matrigel is normal (Figure 4c,d), and their growth curves show exponential growth (for the calculation of the size of cell clusters in Matrigel, please refer to Figures S2 and S3). But for Matrigel droplets of whole tumor cells, the cells in the growth area shrank (Figure 4e) because the average distance of cell clusters decreased.

In contrast, cells migrated to a greater extent toward the center of the Matrigel in the presence of flow (Figure 4f, Video S4). Cells in Matrigel are mobile. They may avoid a bad environment and aggregate slowly, albeit very slowly. The tumor cell cluster remained intact throughout the 14 days of coculture, unlike the close contact with stem cells that would break through the Matrigel interface and migrate outward rapidly (Figure 3). The stem cells are too far away, or the stem cell apoptosis causes the induction effect of stem cells to disappear (Figure 3 and Figure 4a). Microfluidics without precise control cannot realize the ability of stem cells to affect distant tumor cells through EVs and even cannot survive by themselves (Figure 4b–e).

### 2.4. The Interaction between Stem Cells and Tumor Cells in Artificial Cell Nests in a Microfluidic Culture

**Figure 5:**
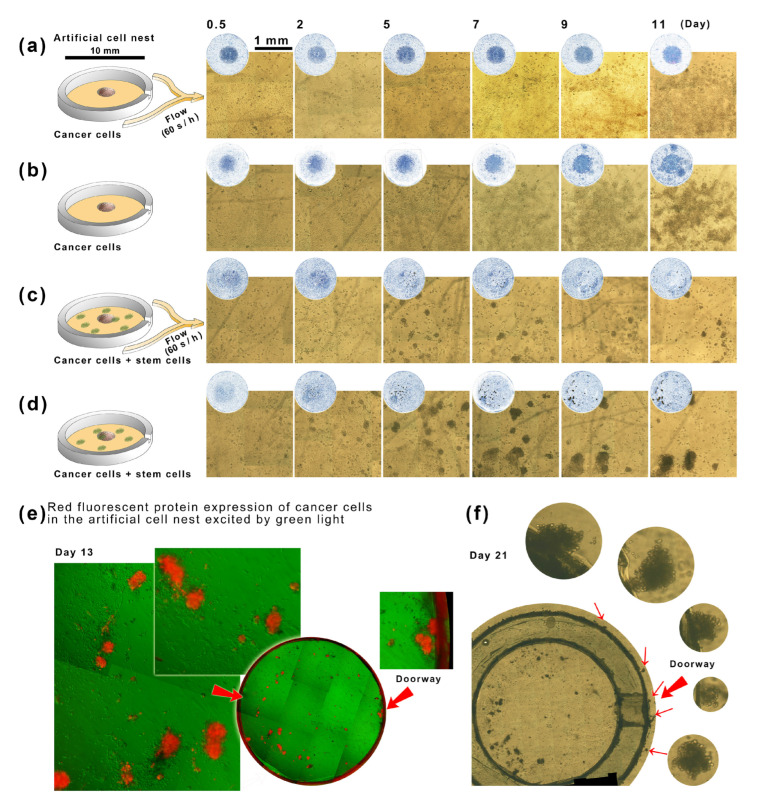
The interaction between stem cells (hiPS) and tumor cells in artificial cell nests: glomeration, spreading, and escaping. (**a**) The transparent artificial cell nest with a 1 mm doorsill (**a**–**d**). We set four experiments, tumor + flow (**a**), tumor without flow (**b**), tumor + stem cells + flow (**c**), and tumor + stem cells without flow (**d**). The stem cells are on the Matrigel and randomly around the mimic tumor. The images of each nest are obtained and pieced together every two days. The circular images show the whole area of the cell nests, and the square shows their detail. (**e**) The red fluorescent cancer cell clusters and their brightfield image with stem cell clones. (**f**) The brightfield images cover the outside area near the gate.

According to the above experimental data, we need to improve in two aspects and re-experiment the cell-to-cell communication between stem cells and tumor cells: (A) control the space to reduce the 30 mm culture dish to 10 mm diameter (the cell nest in microfluidic culture dish, Figure 2h); (B) programmed ultra-slow microcirculation (<40 μm s^−1^, Figure 2h) is realized in this 10 mm cell nest. A smaller coculture space will better maintain the cell microenvironment, and the ultra-slow microcirculation can also achieve EV delivery without destroying the microenvironment. The tumor cells in Matrigel can survive (see Figure 5a). Without microfluidics, tumor cells in Matrigel would stimulate each other more strongly. After day nine, many tumor cells would be migrating out of the boundary of the Matrigel droplet (see Figure 5b). If stem cell clones were seeded randomly within nests (since there were cells everywhere and the borderline of the central tumor cell mass was not clear enough), even with the flow, many tumor cells spheroidized and quickly escaped around. At four weeks (see Figure 5c), the stem cells produced a strong induction of tumor cells. In the absence of flow, the stem cell-induced escape of tumor cells in all directions was more pronounced (Figure 5d, Video S5). Red fluorescent markers showed that the tumor cells originally in the central Matrigel droplet eventually escaped to the surrounding stem cells (Figure 5e) and even outside the gate of the artificial cell nest (Figure 5f). A parallel experiment in which stem cells and tumor cells were cocultured randomly (Figure S4) showed that after 20 days, tumor cells colocalized with the random distribution of stem cells in the microfluidic dish. This phenomenon, from entirely random to colocalization, suggests that the tumor cell cluster will move toward the stem cell cluster until it meets it (Figure S4).

### 2.5. A Long-Distance Induction Experiment of Directed Flow in an Artificial Cell Nest

Stem cells scattered everywhere in artificial cell nests can strongly induce tumor cell spheroidization and scattered migration. Still, this experiment does not adequately utilize the directional ultra-slow microcirculation in artificial cell nests. This stable ultra-slow microcirculatory flow field has a 100–200 μm wide annular microflow and defined internal and external exchanges (Figure 2h,j,m). If stem cells are placed inside the artificial cell nest, away from tumor cell clusters, stem cells will not directly induce tumor cells. Still, they can only induce tumor cells through ultra-slow microcirculation. EVs are the leading carrier of cell-to-cell communication, and the ultra-slow microcirculation in the artificial cell nest will bring EVs to deposit on both sides of the doorway at 5 mm. In this way, the inducers released by EVs will release these inducers away from the stem cells, where the EVs are deposited, and redirect the spheroidization and migration of tumor cells.

**Figure 6:**
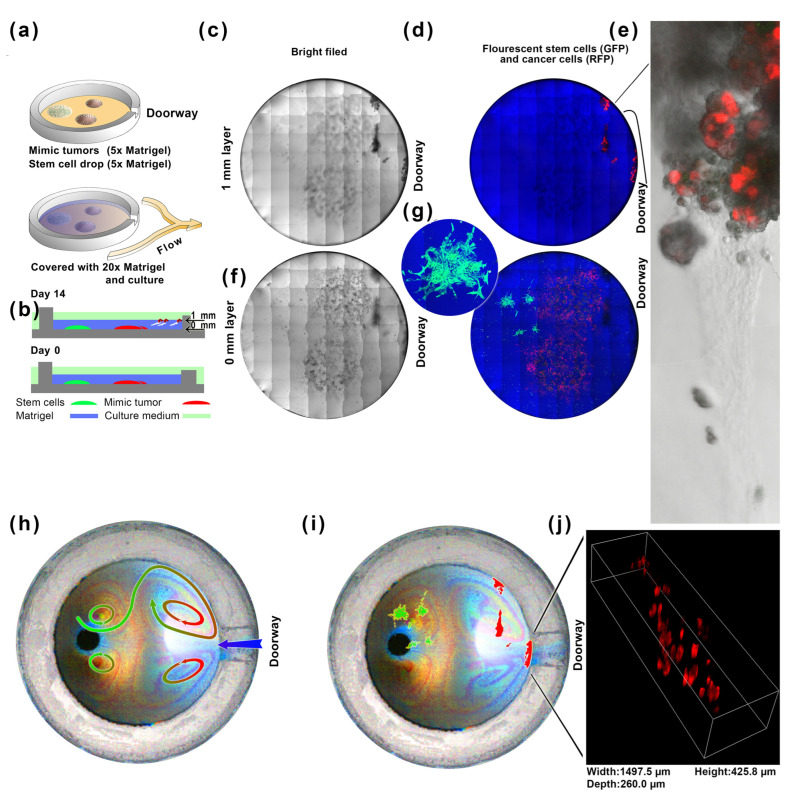
A long-distance induction experiment of directed flow in an artificial cell nest. The flow was adjusted to 60 s per day. There were two mimic tumors (A549-RFP) and one stem cell (embryonic stem cell hESC-H9-eGFP) Matrigel drops inside the cell nest (all at 0mm level), all covered by 20× Matrigel (**a**) to form a 1 mm thick layer (**b**). The confocal microscope scanned the 0 mm and 1 mm layers to obtain brightfield and fluorescence images (**c**–**g**). The insets enlarge the exact stem cell clones (**g**) at the 0 mm layer and the cancer cell clusters at the 1mm layer (**e**). The subfigure (**j**) is a confocal fluorescence 3D image (red fluorescence) of tumor cells (1 mm layer) near to the doorway of the cell nest. The ultra-slow microcirculation is confirmed after the coculture (Video S6). The arrows show the four vortices and the path from the left into the gate (**h**). We overlap the 0 mm and 1 mm images (**i**) to show the cell positions.

The stem cells were mixed with 5× Matrigel and spotted inside the artificial cell nest (Figure 6a). The tumor cells were mixed with 5× Matrigel and sprinkled on the side of the entrance of the artificial cell nest to form a positional relationship of stem cells (inside)–tumor cells (middle)–the EVs deposition area (outer). We covered them with a thin layer of 1 mm 20× Matrigel (Figure 6b), on top of which was the culture medium. The microcirculation program was set to 60 s per day, which means that the EVs released by the stem cells daily will be taken to the EVs deposition area by the ultra-slow microcirculation in the artificial cell nest.

After 14 days, we used a confocal microscope to scan the green fluorescence, red fluorescence, and brightfield images of the bottom (0 mm) and upper (1 mm) parts of the cell nest (Figure 6b) with a confocal laser and stitched them together (Figure 6c–g). After the experiment, we measured the ultra-slow microcirculation in the cell nest for the same culture dish with a 1 mm layer of plastic to mimic the 1 mm Matrigel layer. The flow pattern was superimposed with the confocal images (Figure 6h,i, Video S6) to confirm the ultra-slow microcirculation for the EVs field (Figure 1). The eastern entrance was the area with the highest concentration of nutritional molecule supplements. The north and south sides of the doorway were the two eddy areas with the highest concentrations of EVs from the stem cells (Figure 6h). The remote induction of stem cells was at more than 5mm, and some tumor cells migrated upward in a spherical shape (Figure 6i,j, Video S7). We can see the path of tumor cell spheroids in Matrigel from the picture (Figure 6e). This experiment proves that stem cells can achieve long-range induction of tumor cells through exosomes by ultra-slow microcirculation. (See Figure S5 for the original fluorescence picture or Figure S6 for the original high-resolution fluorescence picture.)

## 3. Discussion

The metastasis [22] occurs through blood circulation [23] and the lymph nodes [24]. The metastasis might be stimulated by the microenvironment [25]. Stem cell niche may promote metastasis [14] or accelerate tumor progression [15,26]. The coculture of lung cancer cells and mesenchymal stem cells produces cellular spheroids [27]. Mesenchymal stem cells abundant in animal bone marrow [15] may promote cancer cell growth [16]. Surgery also accumulates stem cells in the wound, and cancer metastasis has a strong relationship with inflammation [28], for stem cells exist nearly everywhere [17], especially at wound sites [18]. Stem cells may cause epithelial–mesenchymal transition [29]. It is important to realize the experimental simulation of stem cell-to-tumor cell communication. We use the technology of ultra-slow microcirculation cell nests in microfluidic dishes (Figure 1) to study stem-cell-to-tumor-cell communication. The nanoparticles were applied to simulate the flow field of EVs for distant deposition (Figure 2). Although stem cells interact with tumor cells at close range (Figure 3), we explored precise control of programmed flow fields (Figure 4) to achieve exact conditions for coculture. Based on determining that stem cells can significantly induce tumor cell spheres and malignant migration (Figure 5), we have realized through ultra-slow microcirculation technology (Figure 6) that stem cell EVs can cause tumor cell spheres and deterioration at a long distance. This technique is of generally high value for experimental research on stem-to-cell communication. This technology can spatially distance cells–exosomes–cells, and in the future, it may be possible to display changes in exosomes during transport and delivery by labeling exosomes (for example, using immunofluorescence). In this study, ultra-slow microcirculation (~40 μm s^−1^) delivered stem cell exosomes to tumor cells 5 mm away within 1–3 min (daily program setting). Due to the slow flow rate and the short flow duration per day, there is little possibility of fragmentation of exosomes or altered proteomes (further studies are still needed).

## 4. Materials and Methods

### 4.1. The Fluid Drive Technology

The fluid (culture medium) is driven by a magnetically controlled micropump (Figure 1d, oscillator pump, O-pump) [10]. This magnetic O-pump is made of permanent magnetic material with a 1.8 mm–2.3 mm diameter. The vibrator pump is coated with an inert material that is nontoxic to cell or tissue culture. The O-pump is available for order (www.uFDish.com, accessed on 6 March 2023.) or from the author (see Appendix A).

### 4.2. The Drive of Fluid in an Ordinary Culture Dish

The fluid in an ordinary culture dish can be driven by the magnetically controlled micropump controller (Figure 1b) of the microfluidic culture dish by putting the O-pump [10] into the culture medium of the culture dish, and the vibration frequency can be programmed to drive the fluid flow with a specific flow field.

### 4.3. The Microfluidic Culture Dish

Hand-fabricated microfluidic dishes (Figure 1a) [11] or injection-molded-produced microfluidic dishes (Figure 1b,d) can generate a program-controlled directional flow driven by magnetically controlled micropumps. The operation method of this unique microfluidic culture (Figure 1c) is the same as that of a typical culture dish, but there is a C-type cell nest area in the center of the culture dish (Figure 1e). The four-element ultra-slow microcirculation flow field has a stable 1–40 μm s^−1^ flow rate. This area has an actual exchange rate with the outside. Its programmed and precisely controlled flow field can provide cells with regular nutrients and upstream and downstream relationships while maintaining the cell microenvironment (such as the stem cell microenvironment [11]). This microfluidic culture dish system (Figure 1b) is put into a carbon dioxide incubator for a long-time culture (about two weeks). An MP3 player or a PC with a music playlist editor realizes the program flow rate of ultra-slow microcirculation in the central area. Microfluidic culture dish sets are now available for order (see Appendix A).

### 4.4. The Micro Photographing Method of Ultra-Slow Microcirculation

Ultra-slow microcirculation used carbon nanoparticles as an indicator, photographed on a luminescent substrate (Figure 1a), and absorbance values were calculated (see Videos S1, S2 and S6).

### 4.5. The Calculation Method of the Flux into the Artificial Cell Nest

The flow patterns of the light-absorbing substances (nanoparticles or molecular dyes) in the artificial cell nest in the microfluidic culture dish are captured in time lapse as time-series images. Based on the time-series images, the absorbance value of each image pixel of the whole area is calculated, and the half life of the substance escape in the artificial cell nest through the attenuation is obtained by the light absorption curve. Or light-absorbing substances (nanoparticles or molecular dyes) are added outside the artificial cell nest, and the amount of the outside entering the artificial cell nest is calculated (see Figure 2m).

### 4.6. The Cell Growth Method in Matrigel

Typically, cells (including stem cells) are grown on Matrigel. For simulating tumors and accurately locating cell clusters, cells (stem cells or tumor cells) were mixed with Matrigel at 0–4 degrees Celsius (4–10×) and spotted onto the culture disk. After spotting, the cells were placed in a 37 °C incubator for 5–10 min, and then the medium was slowly added to ensure that the cells in the Matrigel did not escape. This study commonly used this spotting method (Figure 3, Figure 4, Figure 5 and Figure 6). Cell growth in Matrigel was monitored daily under a scanning microscope (Figures S2 and S3).

### 4.7. The Image Enhancement Method of Cells in Matrigel

The cells grown in the Matrigel cannot measure the diameter of the spherical cell mass after shooting because they are transparent, and it is not easy to count. An edge detection algorithm was applied to enhance the edges of cells or cell clusters with high contrast to display the position, size, and number of cells or cell clusters (Figures S2 and S3). Suppose it is necessary to show the cell dynamics at different times. In that case, the color channel can be changed so that the images taken at different times can display different colors to visually display the cell dynamics (Figure 3 and Figure 4).

### 4.8. The Extraction Method of Stem Cell Exosomes

The extraction process of stem cell exosomes is complicated. About 200 mL of supernatant after iPSC culture was collected and centrifuged at 300× *g* for 10 min under 4 °C to remove live cells in the supernatant, at 2000× *g* for 10 min to remove cell debris, and at 10,000× *g* for 30 min to remove large vesicles. The supernatant was sterilized by 0.22 μm filtration, and the Amicon Ultra-15 (3 kDa) ultrafiltration tube was balanced with PBS for 4000× *g* centrifugation ultrafiltration twice and 100,000× *g* centrifugation for 70 min twice to obtain exosome precipitates. The exosome pellet was resuspended in 100 μL PBS, transferred to an Ultrafree-CL Filter centrifugal microfiltration tube, sterilized by centrifugal filtration at 5000× *g*, and stored at −80 °C. The shape of exosomes was detected by electron microscopy, CD63 was detected by western blot, and the concentration was quantified by BCA protein. The iPSC exosomes obtained by the above method can be added to the culture medium of lung adenocarcinoma cells (A549), and exosomes at 100 μg mL^−1^ or 200 μg mL^−1^ can significantly promote the growth of A549 cells. Using Transwell (8.0 μm), it can be confirmed that 200 μg mL^−1^ iPSC exosomes substantially enhance the invasion of A549 cells (Figure S1). For the detailed method of the extraction of stem cell exosomes, see Figure S1.

### 4.9. The Cell Lines of Stem Cells and Tumor Cells

There are two stem cell lines, hiPSC-GFP and hESC-H9-eGFP, and one tumor cell line, lung adenocarcinoma cells A549. The main experiments are conducted using hiPSC-GFP, including the extraction of exosomes and the experiments in Figure 3, Figure 4 and Figure 5. The Figure 6 experiment cell line is hESC-H9-eGFP to expand the hiPSC-GFP-related investigations.

## 5. Conclusions

The cell–EVs (exosomes)–cell communication consists of cells that release exosomes, exosome delivery pathways, and cells that receive exosomes (releasing cells and receiving cells can interchange roles or play both roles simultaneously). The most efficient research protocol is to observe and experiment on exosome communication in real time in the co-culture of living cells, which requires the technical ability to add or remove any of these three parts at any time. The current method of extracting exosomes from many cells and interacting with other cells is far from meeting the requirements of this experiment.

(A) The extraction, storage, and feeding process includes many uncertain processes, such as denaturation, degradation, concentration change, and pollution.
(B) Nonreal-time live cell exosome delivery cannot reflect the details of cell–cell interaction feedback.
(C) Manual intervention experiments such as cutting off, reversing direction, and program changes cannot be immediately implemented in the process of cell–cell communication.
(D) These experiments consume a lot of time, samples, and related resources.
(E) Hormonal effects between cells of the same type cannot be ruled out during the culture collection of many cells of the same type.
(F) It is inconvenient to conduct real-time drug intervention cell–cell communication experiments (i.e., related drug development experiments).

However, if the ultra-slow microcirculation method of the microfluidic culture dish is adopted, the above problems in (A–F) can be solved by programmed microflow. For example:

(A) Cocultivation and real-time delivery of exosomes in situ avoid denaturation, degradation, concentration changes, and contamination.
(B) The exosome interaction of living cells during coculture occurs in situ.
(C) Under the condition of ensuring the correct stem cell microenvironment, the programmed flow direction, flow rate, and flow field can realize multiple real-time manual intervention experiments such as cutting off exosome communication, reversing the direction, and resetting the program.
(D) Eliminates many cultured cells and exosome isolation and purification processes, significantly saving time and cost.
(E) Cocultivation and interaction between single-cell clones or small cell clusters, or even single cells can be achieved with solid pertinence and transparent and reliable information transmission logic between cells.
(F) The online coculture of live cells containing exosome delivery direction control can be further combined with drug testing or experimentation, greatly expanding the drug development technology, and improving the accuracy and speed of drug development.

## Data Availability

Data available within the article or its supplementary materials.

## Supplementary Materials

The following supporting information can be downloaded at: https://www.mdpi.com/article/10.3390/ijms241713419/s1.

## Appendix A

**Table A1:** Key Resources.

Reagent or Resource	Source	Identifier
Biological Samples		
A549-RFP cell line	xiangfbio	N/A
hiPSC-GFP	cellapybio	N/A
hESC-H9-eGFP	cellapybio	N/A
human normal liver cell	cellbank	N/A
Medium and reagents		
F-12 Medium	Thermo	SH30023.01
1640 Medium	gibco	N/A
PSCeasy hESC/hiPSC Medium	cellapybio	1001500
Matrigel Matrix	Corning	N/A
Magnetic O-pump	www.ufdish.com/, accessed on 6 March 2023.	N/A
Microfluidic culture dish	www.ufdish.com/, accessed on 6 March 2023.	N/A
