# Supplementary material for: A Microfluidic Experimental Method for Studying Cell-to-Cell Exosome Delivery–Taking Stem Cell–Tumor Cell Interaction as a Case
Source: Int J Mol Sci. 2023 Aug 30;24(17):13419. doi: 10.3390/ijms241713419 (PMC10488205; doi:10.3390/ijms241713419)
Supplement: Supplementary file 1 [file ijms-24-13419-s001.zip › Figures S1 - S5.pdf]

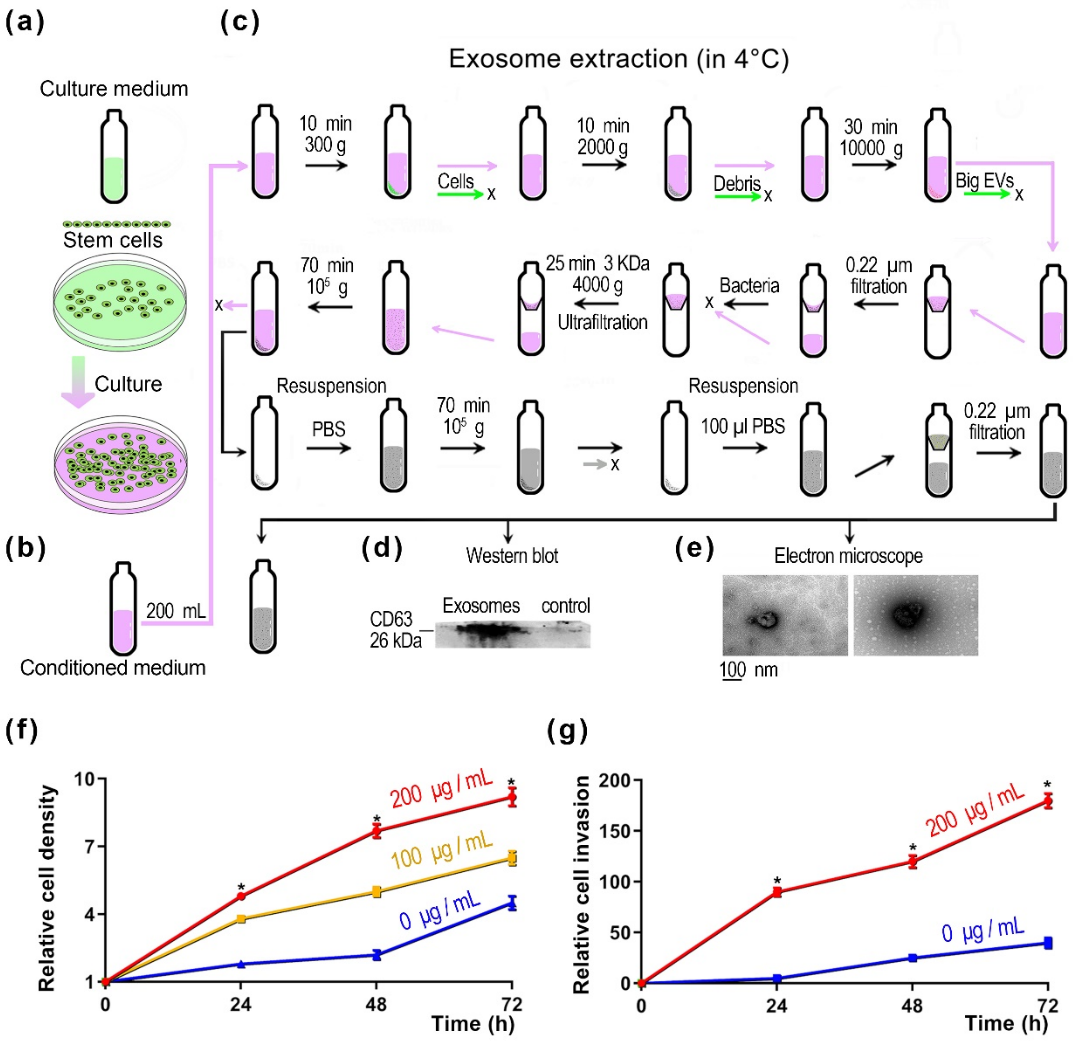

Figure S1 The extraction and detection processes of exosomes.

(a) Stem cells (iPSCs) cultured in medium (mTeSR1).

(b) Collect the medium in which the stem cells have been cultured.

(c) Extraction of exosomes from 200 ml conditioned medium. [300g], [2000g], [4000g], [10000g], [10^5g] and time [min] in the figure represent the acceleration and time of centrifugation. [0.22 μm] indicates a 0.22 μm nuclear pore membrane. [3KDa] means 3Kda ultrafiltration. [PBS] means phosphate buffered solution. [X] means discard.

(d) Electropherogram of exosomal CD63 detected by western blot. Where [Exosomes] means the collected exosome extracts. [Control] is pure culture medium.

(e) Photo of exosomes detected by electron microscopy.

(f) The growth curve of lung adenocarcinoma cells (A549) when different concentrations of exosomes were added to the culture medium. Among them, blue is without exosomes, orange is the final concentration of 100 μg/ml (BCA concentration standard), and red is 200 μg/ml (BCA concentration standard).

(g) Invasion curve determined by Transwell 8.0 μm method (that is, the relative number of cells that A549 is induced to cross below). Among them, the blue color is without adding exosomes, and the red color is 200 μg/ml (BCA concentration standard).

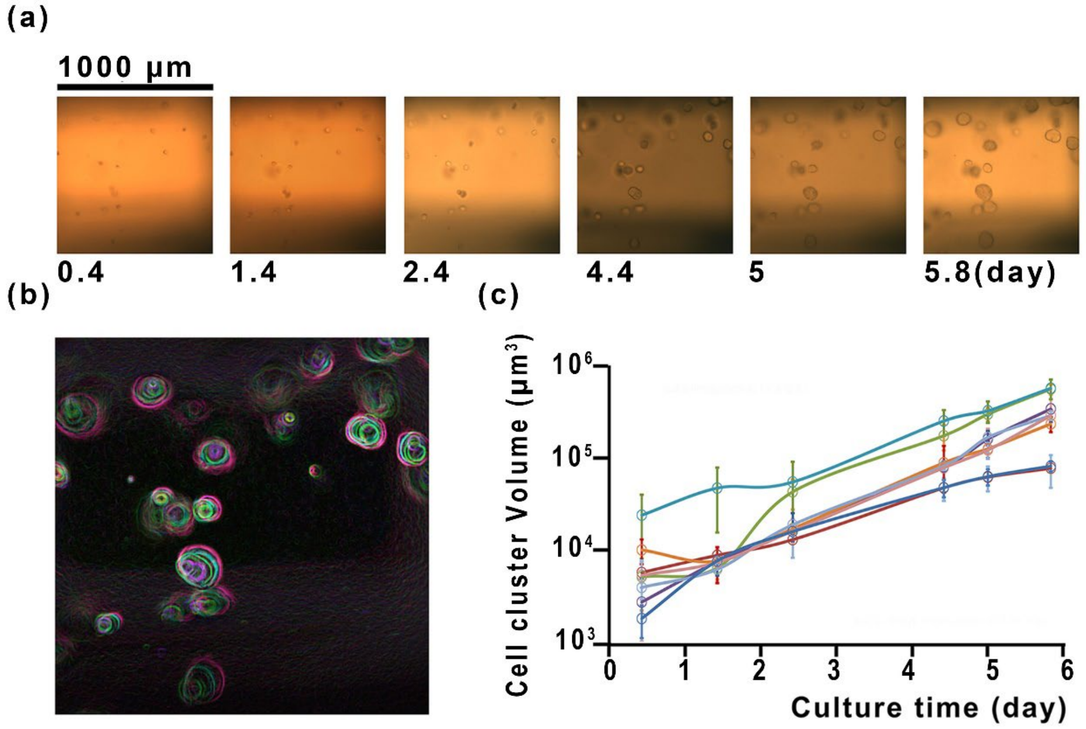

Figure S2 The calculation of the volume of cell clusters grown in Matrigel.

(a) Cell clusters grown in Matrigel (photographed approximately once a day).

(b) Edge detection on micrographs allows for more accurate visualization of cell cluster growth and precise measurements.

(c) The volume change of the cell cluster is calculated according to the diameter (Note: The ordinate is on a logarithmic scale. Error bars are SEM).

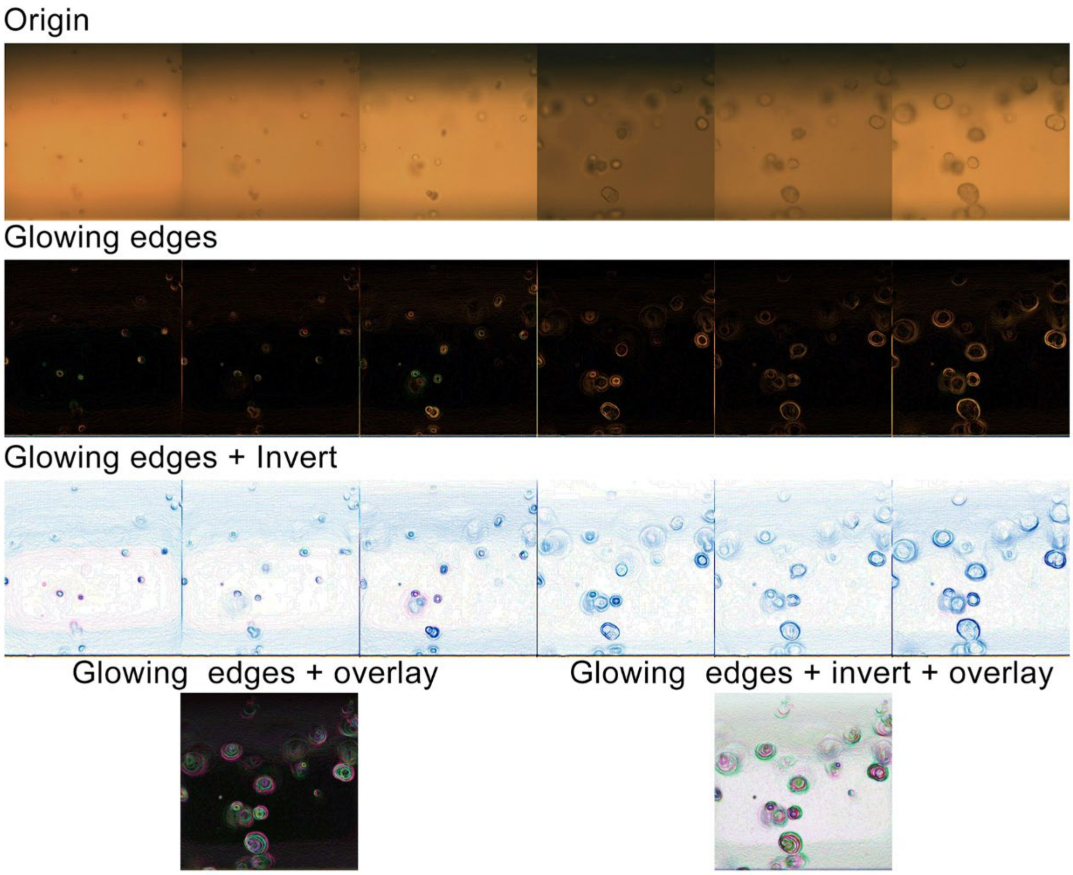

Figure S3 An example of an edge-sharpening image process for cell clusters.

[Origin]: Original image. [Glowing edges], [Glowing edges+Invert], [Glowing edges+Overlay] and [Glowing edges+Invert Overlay]: more transparent images after using functions of the software (Adobe PhotoShop) ([Glowing edges], [Invert], [Overlay]).

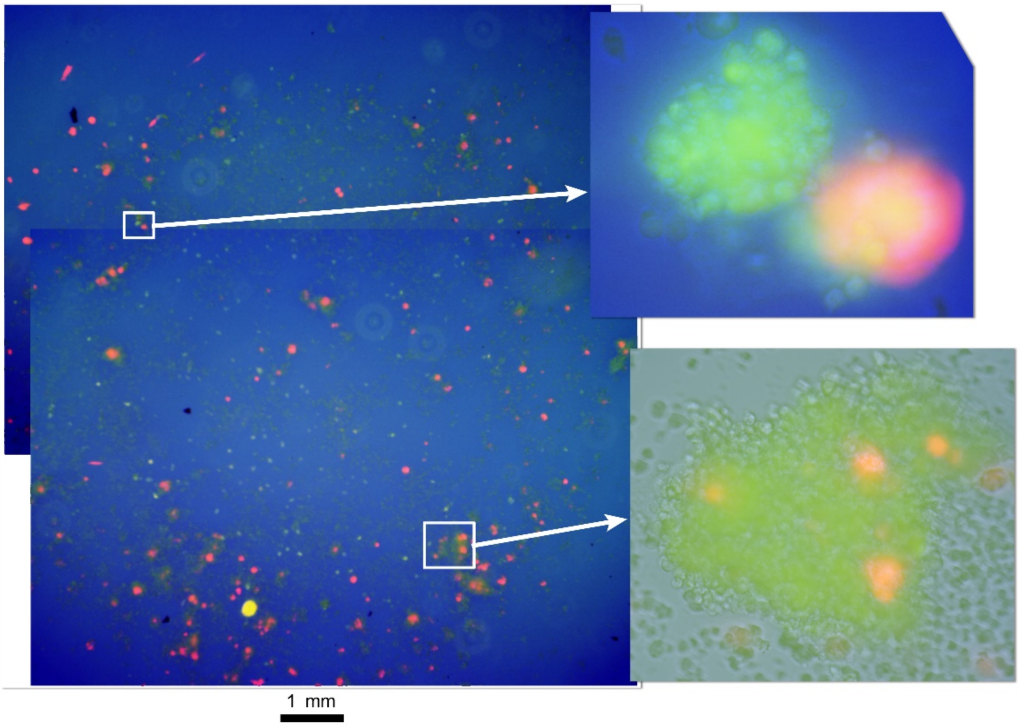

Figure S4 The fluorescent images of stem cells and tumor cells on the 20th day in the microfluidic culture dish (parallel sample of Figure 5 experiment).

The image is in RGB format, where R is red fluorescence (from red fluorescence-labeled tumor cell A549), G is green fluorescence (from green fluorescence-labeled iPSC), and B is a bright field image as a background. The image is stitched from two RBG images. The insets are partial images taken at higher magnifications. During the experiment, the stem cell suspension and the tumor cell suspension were randomly scattered over the microfluidic culture dish with a diameter of 10 mm. After 20 days, the positions of the cancer cell A549 with red fluorescence and the stem cells with green fluorescence overlapped everywhere. The co-localization phenomenon was caused by the getting closer to each other of tumor cell clusters and stem cell clusters during the culture process.

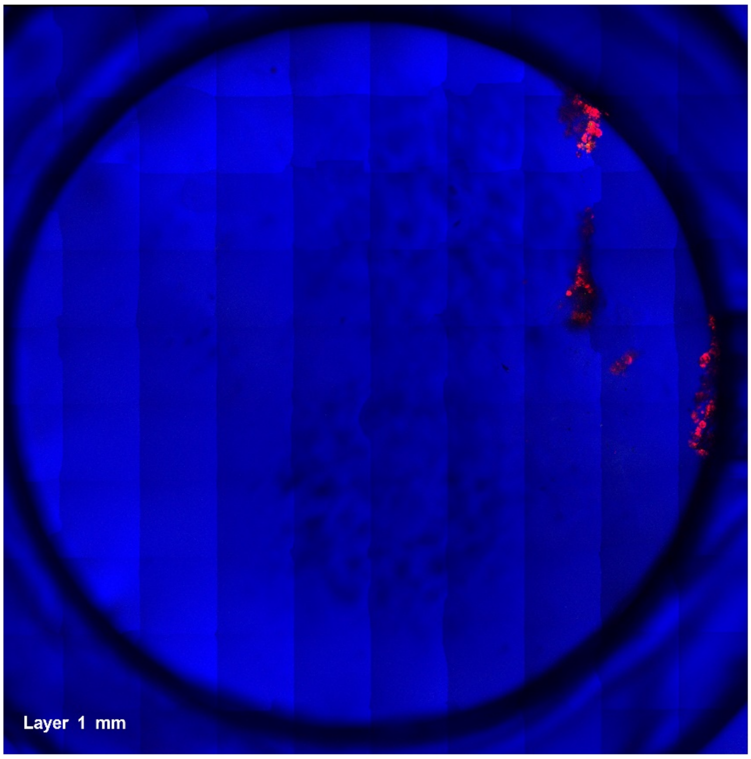

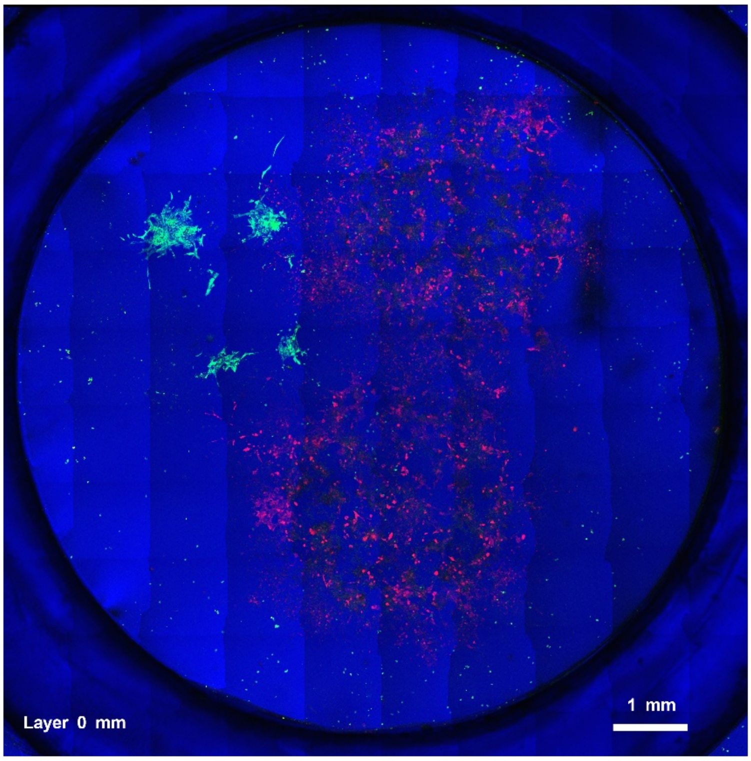

Figure S5 The high-resolution laser confocal full images of the long-distance induction experiment (Figure 5). The high-resolution image is in RGB format (see original big file Figure S6), where R is red fluorescence (from red fluorescence-labeled tumor cell A549), G is green fluorescence (from green fluorescence-labeled iPSC), and B is a bright field image as a background. Each image is automatically stitched from 100 confocal images of 1mm2. The image above (Layer 1mm) is a confocal cross-sectional image at 1mm level from the bottom of the culture dish, and the red fluorescent tumor cell clusters near the doorway (due east) can be seen. The bottom image (Layer 0mm) shows stem cells (green fluorescence) and tumor cells (red fluorescence) in Matrigel at the bottom of the culture dish.
